# Source Reconstruction of Brain Potentials Using Bayesian Model Averaging to Analyze Face Intra-Domain vs. Face-Occupation Cross-Domain Processing

**DOI:** 10.3389/fnint.2018.00012

**Published:** 2018-03-23

**Authors:** Ela I. Olivares, Agustín Lage-Castellanos, María A. Bobes, Jaime Iglesias

**Affiliations:** ^1^Department of Biological and Health Psychology, Faculty of Psychology, Universidad Autónoma de Madrid, Madrid, Spain; ^2^Cognitive Neuroscience Department, Cuban Neuroscience Center, Havana, Cuba; ^3^Department of Cognitive Neuroscience, Faculty of Psychology and Neuroscience, Maastricht University, Maastricht, Netherlands; ^4^Maastricht Brain Imaging Center, Maastricht University, Maastricht, Netherlands; ^5^The Clinical Hospital of Chengdu Brain Science Institute, MOE Key Lab for Neuroinformation, Chinese University of Electronic Science and Technology, Chengdu, China

**Keywords:** brain potentials, ERPs, face recognition, neural sources, N170, N250, N400, P200

## Abstract

We investigated the neural correlates of the access to and retrieval of face structure information in contrast to those concerning the access to and retrieval of person-related verbal information, triggered by faces. We experimentally induced stimulus familiarity via a systematic learning procedure including faces with and without associated verbal information. Then, we recorded event-related potentials (ERPs) in both intra-domain (face-feature) and cross-domain (face-occupation) matching tasks while N400-like responses were elicited by incorrect eyes-eyebrows completions and occupations, respectively. A novel Bayesian source reconstruction approach plus conjunction analysis of group effects revealed that in both cases the generated N170s were of similar amplitude but had different neural origin. Thus, whereas the N170 of faces was associated predominantly to right fusiform and occipital regions (the so-called “Fusiform Face Area”, “FFA” and “Occipital Face Area”, “OFA”, respectively), the N170 of occupations was associated to a bilateral very posterior activity, suggestive of basic perceptual processes. Importantly, the right-sided perceptual P200 and the face-related N250 were evoked exclusively in the intra-domain task, with sources in OFA and extensively in the fusiform region, respectively. Regarding later latencies, the intra-domain N400 seemed to be generated in right posterior brain regions encompassing mainly OFA and, to some extent, the FFA, likely reflecting neural operations triggered by structural incongruities. In turn, the cross-domain N400 was related to more anterior left-sided fusiform and temporal inferior sources, paralleling those described previously for the classic verbal N400. These results support the existence of differentiated neural streams for face structure and person-related verbal processing triggered by faces, which can be activated differentially according to specific task demands.

## Introduction

Person recognition requires the access to and retrieval from long-term memory (LTM) of multi-domain (i.e., facial visual, verbal) information concerning the identity of a known individual (Bruce and Young, 1986). The organization of this diverse information in LTM as well as the neural architecture that supports such cognitive processes have been topics of increasing interest for the neuroscience community in the last few decades. In the present study, we addressed these questions through Event-Related Potentials (ERPs) by contrasting the activity elicited by faces and occupations acting as targets in intra-domain (face-feature) and cross-domain (face-occupation) matching tasks, respectively. Following an experimental paradigm developed by our research group (see for example, Olivares et al., 2000, 2003), the type of knowledge associated to the presented stimuli (realistic drawings of faces) was induced experimentally through several training sessions. Taking into account the exquisite temporal resolution of ERP signals, we have analyzed the neural correlates of the brain activity evoked by recently learned faces with and without associated verbal information by means of both original source-reconstruction approach (Trujillo-Barreto et al., 2004) and statistical methodology in order to analyze the group effects.

A number of face processing models (Bruce and Young, 1986; Burton et al., 1990) suggest that accessing structural and verbal information about known faces occurs at different processing stages. Moreover, some ERP modulations have been proposed in relation with such stages (see for example, Eimer, 2000; Schweinberger and Burton, 2003; Olivares et al., 2015). Thus, the temporal posterior N170 (Bentin et al., 1996) has been identified as a brain response that reflects early face structural processing, likely concerning the generation of face gestalts that further contributes to the identification of individuals (Eimer, 2000). Additionally, there is now substantial evidence that face representations are accessed in the N250 time range (Schweinberger et al., 1995; Kaufmann et al., 2009), following the coding of their configurational properties (see Olivares et al., 2015 for a review).

In relation to later stages of face processing, previous ERP studies on *face recognition* have searched for the correlates of the brain activity related to both intra-domain and cross-domain processing via N400-like tasks. The N400 component was originally associated to verbal processing in language-related research (Kutas and Hillyard, 1980). Modulations of amplitude of this (morphologically) negative ERP have been used as an index of the degree of contextual pre-activation during memory retrieval, or of the amount of post-retrieval integration with the preceding context, thus suggesting associative links between specific contents in LTM (see Debruille, 2007; Kutas and Federmeier, 2000, for reviews). By creating different types of contextual expectancy, several research groups have found N400 priming effects when incongruent faces, occupations or other biographical information were presented as targets following different types of person-related primes (see for example, Barrett and Rugg, 1989; Valdés-Sosa and Bobes, 1990; Debruille et al., 1996; Jemel et al., 1999, 2005; Bentin and Deouell, 2000; Eimer, 2000; Mnatsakanian and Tarkka, 2003; Paller et al., 2003; Boehm and Sommer, 2005; Wiese and Schweinberger, 2008). Most of these studies have used person pair matching tasks to investigate how stored information concerning naturally familiar or highly known people influences the processing of other identities. A few of these studies have evaluated how certain information (i.e., pictorial, face structural, verbal) concerning a unique individual influences the processing of diverse memory contents regarding the same identity. However, in order to disentangle the neurocognitive mechanisms involved in the access to and retrieval of specific types of identity-related information, it is essential to create experimental conditions that directly distinguish those brain responses correlated with the access to and retrieval of face structural information from others related to verbal processing of biographical nature. Furthermore, this would lead to identifying the electrophysiological markers of the processing of different types of knowledge that would be specifically associated to a face seen previously and, ultimately, putative separated neural systems for face structural and face-related verbal processing. In an earlier study (Olivares et al., 2003), we partially dealt with this issue using an original N400-like paradigm which included, among others, an intra-domain matching task (face without eyes followed by the complete face), and a cross-domain matching task (face without eyes followed by the occupation). In both tasks, the stimuli were faces learned without and with associated biographical information, respectively. We found a mismatch response elicited by the face structure that had different timing and topography from those elicited when we combined in the same task both structural and verbal information associated to faces. More specifically, the intra-domain mismatch effect had a shorter duration than the cross-domain one, with the former showing a more right temporal posterior scalp distribution and the latter a mainly occipital location (although we observed in both conditions high voltage scalp values in parietal sites). While it was a first evidence of a nonlinguistic effect analog to N400 but related to face structural information, it remains unclear if the spatio-temporal pattern supporting the processing of the face structure can be differentiated from that concerning the processing of biographical knowledge even when both are triggered by known faces or, alternatively, if they overlap to some extent.

Regarding neural basis for face processing and, of special relevance for the present study, neuroscience studies have identified, based on fMRI and even PET data, the putative cortical network involved in the processing of different types of person-related information (Haxby et al., 2000; Gobbini and Haxby, 2007; Ishai, 2008; Rossion, 2008). Namely, the Fusiform Face Area (FFA), the Occipital Face Area (OFA) and the posterior Superior Temporal Sulcus (pSTS) have been identified as “core” regions in the occipitotemporal cortices that support visual recognition of individuals mediated by face structure (Puce et al., 1996; Kanwisher et al., 1997; Gauthier et al., 2000; Haxby et al., 2000; Kanwisher and Yovel, 2006; Pitcher et al., 2011). The recruitment of such regions by the presentation of a known face might occur together with that of other cortical regions or nodes (frontal, limbic, parahippocampal, anterior, lateral and mesial temporal) more rostrally situated. Such regions are thought to be relevant for the processing of contextual and verbal information that is biographical in nature (Sergent et al., 1992; Gorno-Tempini et al., 1998; Leveroni et al., 2000; Nakamura et al., 2000; Tsukiura et al., 2002; Paller et al., 2003; Fairhall and Ishai, 2007; Rossion, 2008). Importantly, clinical studies concerning modality-specific face recognition disorders (i.e., prosopagnosia) and multimodal people recognition disorders, have contributed substantially to a better understanding of the neuroanatomical sources supporting face processing and people identification. In fact, in an exhaustive review of neuropsychological cases of identity-processing impairment, Gainotti and Marra (2011) stress that the most specific forms of prosopagnosia are due to lesions of a right posterior network, including the OFA and the FFA. In turn, the face identification defects observed in patients with left temporo-occipital lesions are associated to a semantic defect preventing the access to the person-specific verbal information from the visual modality. Moreover, these authors confirm that the face recognition defects derived from right anterior temporal lesions should be considered as part of a multimodal people recognition disorder.

Source reconstruction studies based on scalp-recorded EEG data define the neural origin of the N170 in lateral, basal temporal and extra-striate occipital cortices, including the FFA (Bötzel et al., 1995; Bentin et al., 1996; Kanwisher et al., 1997; McCarthy et al., 1997, 1999; Schweinberger et al., 2002; Itier and Taylor, 2004; Dalrymple et al., 2011). In turn, possible neural generators of N250 have been located in inferior temporal regions (predominantly on the right side), specifically in the FFG, more rostrally than the estimated generators for N170 (Schweinberger et al., 2002; Kaufmann et al., 2009). In relation to the neural sources concerning the classic verbal N400 effect, previous literature has reported the largest neural generators in the left temporal cortex (with a great but lesser contribution of the right temporal cortex) as reflected in brain damaged patients (Olichney et al., 2000), by intracranial recordings (Nobre et al., 1994; Nobre and McCarthy, 1995; Elger et al., 1997; for the fMRI (Friederici et al., 2003) and magnetoencephalographic data (Halgren et al., 2002; Bölte et al., 2010; see Helenius et al., 1998; Kwon et al., 2005; for specific results involving the left auditory cortex; see Van Petten and Luka, 2006; for a review).

The focus of the present study is to compare the spatio-temporal pattern characterizing intra-domain face processing to that for verbal information closely related to known faces, when both are triggered by facial stimuli. According to our primary objective, namely, the search for neurocognitive markers of the access to and retrieval of intra-domain face information and their differentiation from those related to face-related verbal information triggered by faces, we predicted, first, the elicitation of N400-like responses for both incorrect faces and occupations in intra- and cross-domain tasks, respectively. Taking into account previous topographical and neuroimaging data, we anticipated a large increase of activation in occipitotemporal right brain regions for the N400 elicited by faces whereas a more anterior and left lateralized temporal activation would be involved in the N400 elicited by occupations. Additionally, we expected that both modulations and source reconstruction of the earlier N170 and N250 responses provide further evidence for the existence of distinct neural systems for face structural and face-related verbal processing.

To quantify amplitude modulations elicited by the experimental manipulations in the present experiment, we followed a massive univariate approach for repeated-measure ANOVAs for each electrode and each time point. This avoids the traditional biased analysis of prefixed time windows and electrodes accounting for expected ERP modulations, which can miss important experimental effects elsewhere across the entire epoch. However, to control for the increased probability of a type-I error resulting from the simultaneous statistical hypotheses that are being evaluated, in the present study the massive univariate approach has been combined with a correction for multiple comparisons (Lage-Castellanos et al., 2010). Importantly, in order to unravel the neural systems involved in the mismatch effects studied, we used Bayesian Model Averaging (“BMA”, Trujillo-Barreto et al., 2004; Penny et al., 2006). BMA is a recent application of the Bayesian model inference framework (MacKay, 1992) to the solution of the EEG/MEG inverse problem (IP). It has offered optimal results in previous source reconstruction studies on face processing in ERP tasks (Bobes et al., 2010; Olivares et al., 2013), and has shown more detailed source localization, less ghost sources, as well as better highlighting of deep intracranial neural sources than alternative source reconstruction approaches (Trujillo-Barreto et al., 2004). Furthermore, to determine differences in source image maps between conditions, we used a non-parametric conjunction approach (Friston et al., 2005; Nichols et al., 2005; Benjamini and Heller, 2008), which to the best of our knowledge, is the first time it has been used in source analysis of face-related ERPs. This approach was used in order to cope with two long-standing limitations of source reconstruction analyses, namely, the notable inter-subject variability in the scale of Inverse Solution (IS) maps observed and the selection of an adequate threshold for significant voxels in reconstructed images (Genovese et al., 2002). Source reconstruction of the mismatch effects in both tasks would reveal that different neural streams support the access to and retrieval of these distinct types of person-related information.

## Materials and Methods

### Participants

Twenty-eight healthy university students (24 women, mean age 20.7 years, ± 0.6) participated in the experiment as non-paid volunteers. All participants had normal or corrected-to-normal vision and no previous history of neurological or psychiatric diseases. Participants provided written informed consent to participate in the present study. None of the participants was informed about the specific aims of the experiment, which was carried out in accordance with the Code of Ethics of the World Medical Association (Declaration of Helsinki). We also received the approval of the Ethics Committee for Research of the Universidad Autónoma de Madrid CEI-UAM (Reference: CEI-71-1271).

### Stimuli

Forty faces (realistic drawings) served as the learning faces. These faces were created with custom software by combining selected male Caucasian features from an Identikit gallery used in criminological investigations. These faces have been used previously as a learning set in different studies (see for example, Olivares et al., 1999, 2003; Olivares and Iglesias, 2008). Another 320 faces served as mismatch stimuli, that is, faces with different eyes-eyebrows from the original ones, of which 240 were used in the learning sessions (40 faces per session) and 80 in the ERP recording session (for a detailed description of the construction of these faces, see Olivares et al., 2000). The size of each face (presented on a white background 15 cm high × 15 cm wide) on the computer screen was 14 cm high × 10 cm wide (approximately half the natural size). In the recording session, each participant sat 108 cm from the screen, and the faces subtended approximate vertical and horizontal visual angles of 7 and 5.3, respectively.

The face-related verbal stimuli (occupations and names) were obtained by asking 20 judges (average age 28.2 years and with a university-level education) to write down a list of 20 occupations and 20 names that were commonly used in real life. Then, we selected the 40 most repeated items, of which 20 were used for the learning set and the other 20 for the ERP recording session as mismatching targets. The remaining items were used for the forced-choice task of the learning sessions. The associations of faces, occupations and names for learning were random. Word length of occupations varied from 6 to 14 letters (average 8.4 letters) and for names, from four to eight letters (average 5.75 letters). In the recording session, occupations appeared, as in the case of faces, in the center of a white background that was 15 cm high × 15 cm wide, subtending approximate vertical and horizontal visual angles of 0.5 and 2.6, respectively.

### Procedure

#### Learning Sessions

During three consecutive days (six training sessions), each participant was familiarized with two sets of 20 faces each: 20 faces were learned with associated occupations and names, while another 20 faces were learned without associated verbal information. The facial images belonging to the different sets were counterbalanced across conditions. Each learning session was made up of two phases: the *study phase* and the *test phase*, which were carried out separately for each subset of faces.

#### Study and Test Phases for Faces Learned Without Associated Verbal Information

During the *study phase* the participants were required to pay close attention to each one of 20 faces that appeared on the computer screen when they pressed the spacebar of the keyboard. Their task consisted in *memorizing the structure of each face*, paying special attention to the eyes and eyebrows belonging to it and avoiding making verbal associations. The *test phase* consisted in a *forced-choice discrimination task* between matching and mismatching features for each face studied. In this task the participants pressed the spacebar to see each face, which was displayed on the computer screen without the eyes and eyebrows. Simultaneously, below this incomplete face, two numbered combinations of eyes and eyebrows (one of them belonging to the face) were shown. Participants had to decide (and indicate their decision by pressing a particular key) which one completed the face appropriately. Once they had made their choice, the selected combination was superimposed automatically on the face, completing it. Participants could then verify the fit of the selected features on the face and, if not satisfied, rectify their decision. Feedback (by means of a sound from the computer) on mistakes was provided (for a detailed explanation, see Olivares et al., 2000).

#### Study and Test Phases for Faces Learned With Associated Verbal Information

In this case the *study* and *test phases* were quite similar, but participants were required to familiarize themselves, in addition to the faces, with occupations and names associated to each face. In the *study phase*, when a face appeared on the screen, participants pressed the spacebar again so that the labels of an occupation and a proper name, both corresponding to the face presented, were displayed below it (for example, Doctor-Peter). In the *test phase* the participants carried out the same *forced-choice discrimination task* for the faces studied (with incomplete faces with eyes-eyebrows below), but were also asked (after the face completion) to recognize, between two alternatives said by the experimenter, both the occupation and the name that had been presented previously (in the study phase) as associated with the displayed face. The experimenter recorded these verbal reports. The order of training for each subset of faces was alternated from one session to the next one.

In order to evaluate the learning progress, performance measures were taken in each session for each learning set. We used the *d’* (*discrimination sensitivity*, Swets, 1964) as an index of the participant’ ability to differentiate progressively match from mismatch facial features for face-feature associations. The formula used to calculate the *d’* was *d’*= 0.6 log [(pH (1-pF))/(pF (1-pH))], described in Meyer et al. (1988), where pH denotes the mean probability of hits (correctly selected features) and pF the mean probability of false alarms (incorrectly selected features). In the case of occupations, the total number of items recalled in each session was the learning measure used (since the names were not used in the cross-domain task analyzed in the present study, the results related to them will be not reported).

#### ERP Recording Session

**Figure 1:**
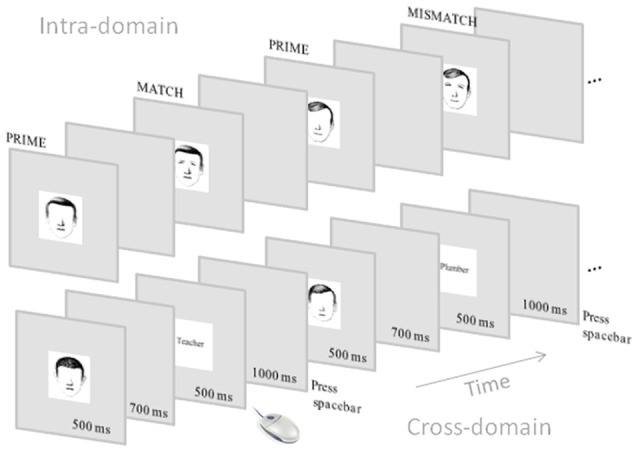
Schematic representation of the event-related potential (ERP) tasks. Note that the intra-domain task was carried out with faces without associated verbal information whereas the cross-domain task was carried out with faces with associated occupations.

The ERP recording session was carried out 3 days after the end of the learning sessions. At the beginning of the ERP session, participants reviewed both sets of learned faces in a similar way to that used in the study phase of the learning sessions. For ERP recording, participants carried out different matching tasks concerning the learned information in which the presentation order varied from one participant to the next one. For the purpose of the present study we analyzed data concerning the intra-domain task, which consisted of face-feature matching, and the cross-domain task, which consisted of face-occupation matching. For the intra-domain task, the stimuli used were those 20 faces learned without associated verbal information. Each one of these faces was displayed (after the participant key press) as prime stimulus without the eyes-eyebrows for 500 ms and the screen went black for 700 ms. In half of the trials (40), each target face was then displayed automatically with the match features belonging to the face. In the other half, the targets had mismatch features. The complete face was displayed for 500 ms (+1000 ms post-stimulus) and the participant had to decide whether the completion was the correct one or not by using the two mouse keys. For the cross-domain task, the prime stimuli were also incomplete faces but belonging to the set of faces learned with associated verbal information. Note that the structure of these faces was learned using the same procedure as in the other set of faces, thus we kept the prime conditions in both ERP tasks as similar as possible. The target stimuli were either match or mismatch occupations according to what the participants had learned in the learning sessions (Figure 1). In both tasks, the presentation of match and mismatch targets was randomized. Reaction times were recorded in each task as well as the *d’* (in this case as an index of participant’s ability for discriminating match from mismatch targets).

### EEG Recording

We recorded the EEG using a MEDICID 5 (I.C. Neuronic, S.L.) with 60 Ag/AgCl electrodes mounted (according to the extended 10/20 International System) on a Neuroscan QuickCap (from anterior to posterior and left to right positions, the 60 recording sites were: FP1, FPz, FP2, AF3, AF4, F7, F5, F3, F1, Fz, F2, F4, F6, F8, FT7, FC5, FC3, FC1, FCz, FC2, FC4, FC6, FT8, T7, C5, C3, C1, Cz, C2, C4, C6, T8, TP7, CP5, CP3, CP1, CPz, CP2, CP4, CP6, TP8, P7, P5, P3, P1, Pz, P2, P4, P6, P8, PO7, PO5, PO3, POz, PO4, PO6, PO8, O1, Oz and O2) and the tip of the nose was used as the reference. EOG was recorded from electrodes placed just above the left supra-orbital ridge (vertical EOG) and on the left outer canthus (horizontal EOG). Impedance was kept usually below 5 kΩ and always below 10 kΩ, and in any case with an appropriate signal/noise ratio for high-density recordings standards. EEG and EOG signals were filtered on-line between 0.5 Hz and 100 Hz (3 dB down). We computed ERPs off-line, averaging segments of 256 points of digital EEG (12-bit A/D Converter, sampling rate of 250 Hz). The segmentation was synchronized with the target stimuli, that is, complete faces and occupations in the intra- and cross-domain tasks, respectively. These segments covered 1000 ms, comprising a pre-stimulus interval of 200 ms (for baseline correction). Additionally, DC shifts were corrected using linear detrending. Before averaging, individual EEG segments were visually inspected and those with excessive EOG artifacts or voltage fluctuations (±100 μV) were excluded from further processing. After visual inspection, the number of segments for averaging was equalized across conditions. ERP averaging was based on a minimum of 24 segments out of 40 total trials per condition (30 on average for faces and 29 for occupations).

### ERP Analysis

As pointed out in the “Introduction” section, in order to avoid the *a priori* selection of the latencies and electrodes of interest, we used a massive univariate repeated measures ANOVA. This approach requires the computation of a two-way repeated measures ANOVA model for each electrode and each time point to establish differences in the amplitudes of the ERPs between the levels of the two studied factors: TASK, with two levels (intra- and cross-domain) and MATCH, with two levels (match and mismatch). No sphericity correction was required since this is a 2 × 2 factorial design. This approach leads to a multiple comparison problem due to the simultaneous ANOVA across all electrodes and latencies. Therefore, the increased risk of type I error was controlled for with False Discovery Rate (FDR) statistics (Genovese et al., 2002). The selection of the threshold was assessed for controlling the FDR at level *q* = 0.05 (Genovese et al., 2002; Lage-Castellanos et al., 2010). To determine which differences are responsible for the rejection of the omnibus null hypothesis, those electrodes and time points that resulted significantly at the ANOVA after FDR correction were submitted to *post hoc* analyses. At the *post hoc* tests, the FDR correction controls the multiplicity of comparisons within-factor, and the multiplicity of electrodes and latencies. We present the results in terms of spatio-temporal maps of significant effects.

### Source Analysis

#### Source Modeling

For the modeling of the neural generators associated with the electrophysiological effects elicited by the mismatch targets, we used the BMA approach (Trujillo-Barreto et al., 2004; Penny et al., 2006). BMA is an application of the Bayesian model inference framework to the estimation of the primary current densities (PCDs) inside the brain given the scalp recorded data; i.e., to the solution of the so called EEG/MEG IP, under the evidence approximation (MacKay, 1992). It allows the combination of several inverse solutions into a single one by means of some kind of weighted average that accounts for any uncertainties we might have about any (or all) of the specific inverse solutions under consideration. The weights in this average are probabilities that express the support that each of the competing models (i.e., solutions) receives from the data (i.e., we let the data choose the best group of models automatically) in terms of goodness-of-fit and complexity of the model (number of parameters or sources to be estimated). That is, the best models will be the ones that can fit the data best while using the lowest number of parameters. With this approach instead of choosing a single inverse solution from those under consideration, all solutions are used, but their influence on the final average solution is weighted. In the present study, the different models (inverse solutions) to be averaged out for a given data set were created by finding LORETA inverse solutions (Pascual-Marqui et al., 1994; Pascual-Marqui, 2002) under different anatomical constraints, that is, different parts of the brain. These constraints resulted from the average Probabilistic MRI brain atlas created at the Montreal Neurological Institute (MNI Brain, Evans et al., 1993, 1994; Collins et al., 1994; Mazziotta et al., 1995). BMA is able to assign higher probability to the LORETA solutions that are confined to those brain areas which receive the highest support from the data, identifying those brain regions that actually contribute to the generation of the recorded EEG. Likewise, brain areas that receive poor support from the data are pruned from the final BMA solution automatically. This BMA approach has demonstrated to provide more robust source estimates than any of the individual LORETA solutions considered (see Trujillo-Barreto et al., 2004, for a description of the mathematics and properties of the BMA approach).

The intracerebral PCDs were estimated over a source space (grids) of 6000 triangles resulting in 5656 generators, constrained to 76 anatomical compartments of the cortical surface, which were chosen from the MNI Brain. With this information, the physical term (electric lead field) that relates the intracerebral activity to the scalp electric fields was computed. The forward model used in this case consisted of three spheres modeling piecewise homogenous compartments: brain, skull and scalp. The conductivity values selected in our case were 0.33, 0.022 and 0.013 Ω/m for the brain, scalp and skull, respectively (Oostendorp et al., 2000; Zhang et al., 2006).

Voltage values submitted to source reconstruction corresponded to the individual average amplitude of the time interval 330–440 ms in the mismatch ERPs (the same interval for all participants). In this time window, we observed the most conspicuous mismatch effect in both tasks as well as a constant topographical distribution in the voltage scalp maps. Furthermore, it was preliminarily evaluated with permutation tests (Blair and Karniski, 1993; Nichols and Holmes, 2002) and corroborated by massive univariate repeated measure ANOVAs. Importantly, face-sensitive components were also submitted to source modeling using the amplitudes of well-defined peaks from each participant that were observed in each task, namely, N170 for both intra- and cross-domain tasks as well as P200 and N250 for the intra-domain task.

#### Second-Level Group Analysis of Source Reconstruction Maps

To determine differences in source-reconstructed images between conditions we used a conjunction analysis (Friston et al., 2005; Nichols et al., 2005; Benjamini and Heller, 2008). This strategy can handle the differences in the scale of individual IS maps, and the uncertainty about the null distribution of IS coefficients, two unresolved problems in IS analyses. Initially, for each participant, the generators that would provide a relevant contribution to the IS map were determined using a *mixture density model* which classifies the generators into a null (non-active) and an alternative (active) distribution using the local FDR algorithm (Efron, 2004). FDR is an empirical Bayes version of the standard FDR methodology (Benjamini and Hochberg, 1995) for large-scale simultaneous hypothesis testing, which focuses on densities rather than tail areas of the distributions. It permits the empirical estimation of a null hypothesis distribution. After this step, individual maps were considered in a binary form, defining those voxels corresponding to the alternative distribution (active generators) with 1. These binary maps are invariant to the scale differences in IS maps across participants, since binary variables (0,1) are independent of the scale of the generator’s coefficients. Next, conjunction maps were computed for each of the ERP components that were determined at the massive univariate repeated measures ANOVA analysis: N170, P200, N250 and N400. The conjunction is computed as the proportion of participants having a particular voxel active (1 in binary form) and reported in a (0,1) scale.

After that, we addressed the statistical question of comparing binary maps of the same participant across the different levels of the studied factors. Note that the relevant differences to study in this design are the differences in activation of maps between the levels of the factor TASK (intra-domain and cross-domain) for each participant. Consequently, for each participant, the binary maps corresponding to the compared conditions were subtracted, obtaining individual maps with possible values of −1, 0 and 1. The voxels having zeros indicate the same category (active or non-active in the binary map) in the two compared conditions whereas the non-zero voxels indicate a difference between maps at this particular voxel (the sign indicates the direction of this difference). Then the conjunction of the subtraction maps was computed across participants. The conjunction at each voxel is calculated as the proportion of participants having this voxel active at the subtraction maps, considering the sign of the difference.

The statistical threshold of conjunction maps was computed using permutation tests with 10,000 iterations, under the null hypothesis of the equality of experimental conditions. This null hypothesis implies that the statistics used for assessing the conjunction null hypothesis randomly fluctuates around zero for each voxel. The p-values for permutation tests were thresholded using FDR at *q* = 0.05. Next, the conjunction maps for the difference between conditions were displayed presenting only the voxels having conjunction values above the estimated threshold.

## Results

### Behavior

#### Learning Sessions

In order to compare the learning progression between the two subsets of faces, we carried out a two-way repeated-measure ANOVA using FACE SUBSET (two levels) and SESSION (six levels) as the main factors. The analysis showed that SESSION was significant (*F*_(3,94)_ = 43.3, *p* < 0.0001) but there were not significant differences for the factor *face subset* (*p* = 0.06) nor for the interaction FACE SUBSET × SESSION (*p* = 0.3). In the case of occupations, the number of correctly recognized items also progressively increased in consecutive learning sessions (*F*_(3,76)_ = 7.6, *p* < 0.0001). Average number of recognized items ranged from 19.3 in the first session to 19.96 in the last session.

#### ERP Recording Session

In the recording session the *d’* values obtained in the intra-domain and the cross-domain matching tasks showed that the participants optimally discriminated match from mismatch targets in both cases. The *d’* in the intra-domain task was 1.59 (with corresponding hit and false alarms probabilities of 0.91 and 0.11, respectively) and in the cross-domain task it was 1.61 (with corresponding hit and false alarms probabilities of 0.91 and 0.07, respectively). T-test confirmed that both values were not significantly different. The ANOVA carried out to compare the reaction times of the different experimental conditions showed that only the factor MATCH was significant (*F*_(1,27)_ = 8.09, *p* = 0.008), with RTs for match targets (mean: 876.5 ms) being faster than RTs for mismatch targets (mean: 901.15 ms). The interaction TASK × MATCH was not significant.

### Electrophysiology

**Figure 2:**
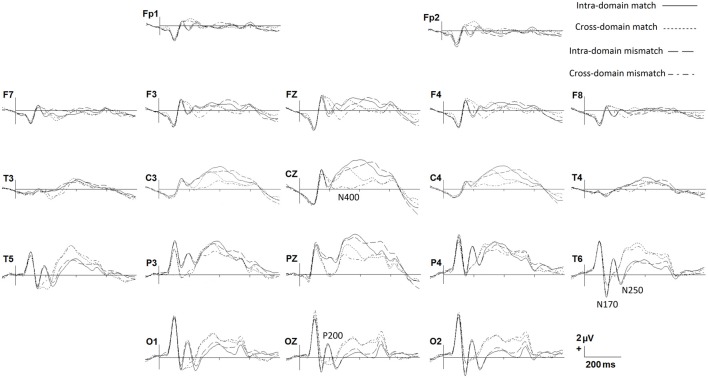
Grand average ERPs for each experimental condition. Only some representative recording sites are shown.

In Figure 2 we show grand average ERPs elicited in the two tasks by both match and mismatch targets. Visual inspection indicates notable differences between tasks around 200 ms after stimulus onset, where a prominent positive peak in posterior regions, namely, P200, is present exclusively for faces. A negative peak around 270 ms, which would correspond to the modulation of the N250 component, is also present solely for faces in the posterior regions. Also of note, between around 300 and 600 ms match ERPs show, in general, more positive amplitudes than mismatch ERPs, mainly in central regions, denoting a N400-like effect in both tasks. This effect seems to be more conspicuous in the cross-domain task. In the N170 window, faces seem to elicit slightly larger negative amplitudes than occupations.

**Figure 3:**
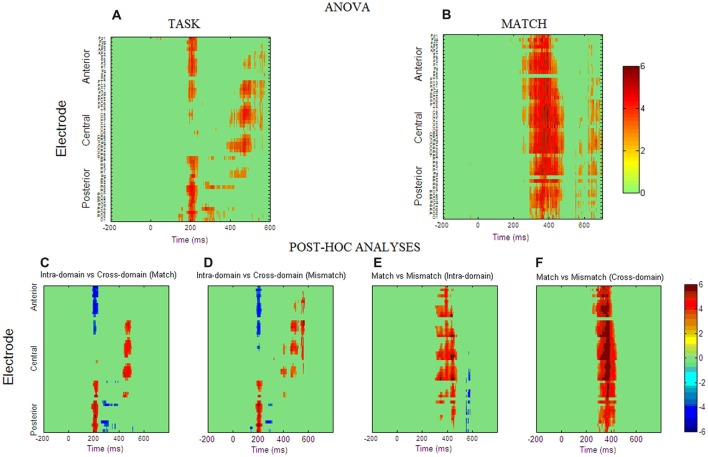
Spatio-temporal maps obtained from repeated measures ANOVA analysis for factor TASK **(A)** and factor MATCH **(B)**. The ANOVA *p*-values were transformed to Z scores for clarity of interpretation. The Z statistics of those space-time points that fulfill the false discovery rate (FDR) criterion are presented in a color scale. The interaction between the two factors was not significant for any space-time point. **(C–F)** Spatio-temporal maps obtained from *post hoc* test computed at those space-time points where the main effects were reported as significant. The maps are presented in t-statistic and were thresholded with the FDR criterion (*q* = 0.05).

Figures 3A,B show spatio-temporal maps derived from repeated-measures ANOVAs carried out to quantify these observed effects in each recording site and each time point, using TASK and MATCH as factors. According to these analyses, the main effect of TASK (Figure 3A) is present around 200 ms post-stimulus onset in both anterior (from 184 ms to 232 ms) and posterior (from 180 ms to 236 ms) regions. This effect was also significant around 400–600 ms, confined mainly to central regions, and around 300 ms to posterior ones. *Post hoc* comparisons (Figures 3C,D) indicated that, in general, ERPs elicited by faces around 200 ms were more positive than those elicited by occupations in posterior regions, with the opposite effect in anterior regions, denoting the presence of a P200 component in the case of facial stimuli. In the N250 time-window ERPs for occupations were significantly more positive than for faces, also showing that this component was elicited essentially by facial stimuli. The *post hoc* comparisons in the later latencies suggest that, in general, responses to faces were more positive than to occupations between 400 ms and 600 ms at central sites.

For the MATCH factor (Figure 3B) the results reflected that match and mismatch ERPs differ significantly between 300 ms and 450 ms and these differences were widely distributed across the scalp. *Post hoc* comparisons indicated that in both tasks match ERPs are significantly more positive than mismatch ERPs in these latencies, although the occupations seem to have the largest effect, as suggested by spatio-temporal statistical maps derived from the *post hoc* comparisons (Figures 3E,F). Interaction TASK × MATCH was not significant in any time point or electrode.

### Source Analysis

**Figure 4:**
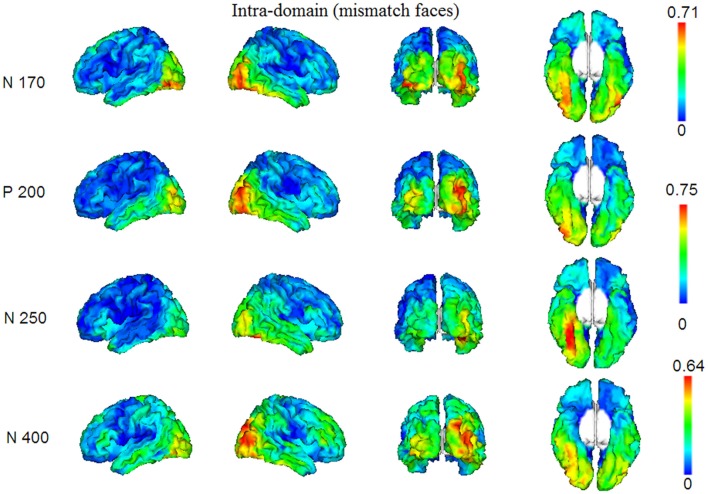
Group conjunction maps (the proportion of participants that presented activation at each voxel) of the source images corresponding to the studied ERP effects elicited by the mismatching targets in the intra-domain task.

**Figure 5:**
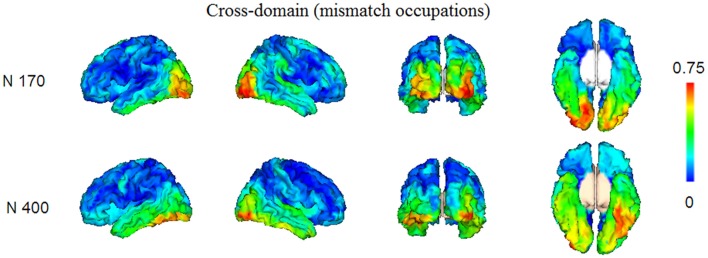
Group conjunction maps (the proportion of participants that presented activation at each voxel) of the source images corresponding to the studied ERP effects elicited by the mismatching targets in the cross-domain task.

In Figures 4, 5, we show the source images obtained in the group of participants corresponding to the analyzed effects in both intra-domain (N170, P200, N250, N400) and cross-domain (N170, N400) tasks. Table 1 shows a summary of those cortical regions that presented the largest number of voxels with the maximal conjunction values (proportion of participants that presented activation at each voxel) in each effect and task.

**Table 1:** Summary of the conjunction analysis carried out on ISs maps from mismatching targets in each task.

	Effect	Cortical region	Cluster size (# voxels)	Conjunction value (SD)
FACES	N170	Fusiform R	59	0.58 (0.04)
		Occipital Middle R	49	0.59 (0.05)
		Occipital Inferior L	38	0.62 (0.05)
		Occipital Inferior R	36	0.59 (0.05)
	P200	Occipital Middle R	67	0.65 (0.06)
		Occipital Middle L	45	0.56 (0.03)
		Occipital Inferior R	39	0.64 (0.05)
	N250	Fusiform R	66	0.66 (0.07)
		ParaHippoc. R	10	0.60 (0.03)
	N400	Occipital Middle R	66	0.54 (0.05)
		Occipital Inferior R	39	0.50 (0.03)
		Occipital Sup. R	38	0.55 (0.06)
		Fusiform R	37	0.50 (0.03)
OCCUPATIONS	N170	Occipital Middle L	59	0.60 (0.02)
		Lingual R	59	0.65 (0.05)
		Lingual L	42	0.61 (0.03)
	N400	Fusiform L	57	0.63 (0.06)
		Temporal Inferior L	40	0.60 (0.04)
		Occipital Inferior L	38	0.62 (0.03)
		Occipital Inferior R	36	0.63 (0.07)
FACES vs. OCCUPATIONS	N170: F > O	Fusiform R	19	0.60 (0.06)
	O > F	Lingual R	51	0.65 (0.04)
	N400: F > O	Occipital Sup. R	37	0.55 (0.06)
	O > F	Fusiform L	49	0.64 (0.05)
		Temporal Inferior L	36	0.60 (0.04)

*Cortical regions with the largest clusters and conjunction values in each analyzed ERP effect. F, Faces; O, Occupations; L, Left; R, Right. The results of the statistical analysis using a permutation test to compare estimated neural sources of those effects elicited in both tasks are displayed at the bottom*.

#### Intra-Domain Task

In this task, reconstructed sources corresponded to the N170, P200, N250 and N400 components. The N170 elicited by faces in the intra-domain task appears to originate from a more extended source (higher number of voxels) across participants in the right fusiform cortex, with a somewhat less extended source in the right middle occipital cortex and with important bilateral involvement of the inferior occipital region. The P200 sources were located in very posterior regions, predominantly in the right middle occipital cortex, but with notable involvement of the left middle and the right inferior occipital cortices. In the case of the N250, the predominant source was extensively located in the right fusiform region, with some contribution of the right parahippocampal cortex. In the N400 window, the sources were notably right-sided and predominantly occipital, with notable involvement of the right fusiform region.

#### Cross-Domain Task

Reconstructed sources were those relative to the N170 and N400, which were the only ERP components of relevance observed in this task. The sources of the N170, elicited by occupations in this task, were predominantly located in very posterior regions, including the left middle occipital and bilateral lingual cortices. In turn, in the case of the N400, the most prominent sources were located in left fusiform and left inferior temporal regions and bilaterally in occipital inferior regions.

#### Comparison of the N170 and N400 Effects Elicited in Both Tasks

**Figure 6:**
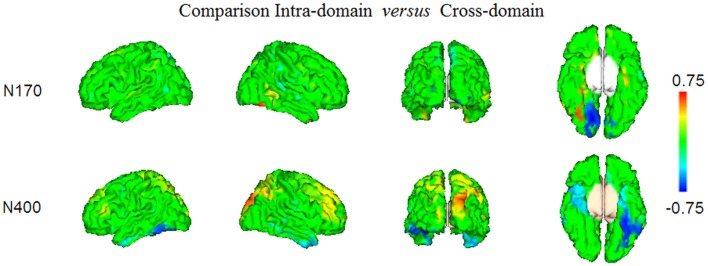
The results of the statistical comparison using permutation tests of the maps presented in Figures 4, 5, are shown. Only the voxels where the comparison was significant at FDR level *q* = 0.05, are displayed. Note that positive conjunction values correspond to those regions where the effects elicited in the intra-domain task were larger than in the cross-domain task, whereas the negative conjunction values represent the regions in which there was comparatively larger activity in the cross-domain task.

In Figure 6 and Table 1 (bottom), we show the results from the statistical comparisons (using permutation tests) of the conjunction maps for N170 and N400 from each task. According to such analyses, the N170 for faces showed larger conjunction value and number of activated voxels in the right fusiform region than the N170 elicited by occupations, whereas the latter elicited comparatively larger values in the right lingual region. In relation to the N400 effect, the analyses showed that faces showed greater conjunction values and number of active voxels in the right superior occipital cortex, whereas the left fusiform and the left inferior temporal cortices were the most largely activated regions in the case of N400 for occupations when compared with the N400 for faces.

## Discussion

### Shorter RTs Associated to the Correct Target Conditions Show the Expected Mismatch Effect in Our Experiment

In our search for neurocognitive markers of face structural processing in contrast to that of face-related verbal processing triggered by faces, we thoroughly trained our participants with different face-related information and analyzed the brain activity elicited in two different N400-like tasks concerning the access and retrieval of intra-domain and cross-domain information. The learning progression and the good performance in the ERP recording session (as indexed by increasing and high *d’* values, respectively) showed that our participants reached an optimal acquisition of identity-related information and hence adequate familiarization with the two studied sets of faces, that is, faces with and without associated verbal information. Likewise, the similar *d’* and RTs values obtained in the two ERP recording tasks indicated that intra-domain and cross-domain processing entailed the same level of difficulty for our participants. In turn, the comparison of RTs between match and mismatch targets revealed the classical significant difference between both types of targets in matching tasks.

### Larger Negativities for Mismatch Targets Reflect a Sort of N400 Effect in Both Intra- and Cross-Domain Tasks

In line with the differences observed in RTs between match and mismatch targets, we found the largest amplitudes of N400-like potentials elicited by mismatch targets when compared with match ones in both tasks. This effect replicates previous findings of lowest positive-going voltage values around 300–600 ms associated to mismatch targets in face-related paradigms (Barrett and Rugg, 1989; Jemel et al., 1999; Bentin and Deouell, 2000; Eimer, 2000; Wiese and Schweinberger, 2008; Herzmann and Sommer, 2010; Olivares and Iglesias, 2010). The amplitude effect found in the case of faces in the intra-domain task supports the existence of a N400-like potential which might be related to face structural processing (Olivares et al., 2003). Furthermore, non-significant behavioral differences were observed between the two tasks. The larger MATCH effect in the cross-domain task (as suggested by spatio-temporal statistical maps in Figures 3E,F) could be due to the fact that the targets (i.e., occupations) in this task belong to a naturally wider verbal category than the targets (20 original artificial faces) in the intra-domain task. According to the “N400 knowledge inhibition hypothesis”, the amplitude of this ERP depends on the amount of knowledge that is inhibited and on the strength of its previous activation: the stronger the activation, the greater the required inhibition and the greater the N400 amplitude (Debruille et al., 1996; Debruille, 2007). Thus, in our experiment, occupations would activate a greater number of verbal neighbors when compared to the recently known artificial faces. Accordingly, the set of preactivated occupations would require a greater neural inhibition with the arrival of the mismatching target and would thus elicit the relatively larger N400 amplitude. Alternatively, amplitude modulations around these latencies might be influenced by the access to verbal information concerning faces (Eimer, 2000; Schweinberger and Burton, 2003), which raises the question of the domain specificity of these responses, an issue of continuous debate in the neuroscientific literature.

### Neural Activity Associated to N170s for Faces and Occupations Might Reflect Different Cognitive Operations in Initial Visual Processing

Source reconstruction of the N170 component also suggests a neurofunctional distinction between intra-domain and cross-domain processing in the present experiment. The neural sources of the intra-domain N170 found here encompass mainly the face-selective FFA and OFA regions, although we have also observed a certain degree of contribution of the left inferior occipital region. These regions partially correspond with those reported in previous literature concerning source modeling of N170 with MEG (Halgren et al., 2000; Itier et al., 2006; Taylor et al., 2011). Itier et al. (2006), for instance, found a bilateral posterior and a simultaneously active right lateralized ventral source around the fusiform gyrus in the N170 time-window. Rossion et al. (2003), who used a dipole fitting approach for source analysis in ERPs, reported that N170 was associated to two dipolar sources located in the right lateral inferior occipital cortex/posterior fusiform gyrus. Additionally, Corrigan et al. (2009) found that face-processing and N170 sensitive activity, measured by both fMRI and ERP source modeling with LORETA (Pascual-Marqui et al., 1994), overlapped in a number of cortical areas, including bilateral fusiform gyri, right superior, middle and inferior temporal gyri and even the bilateral precuneus. The fact that N400 and N170 for faces have a similar pattern of reconstructed sources (i.e., large involvement of OFA and FFA areas) in the present study indicates that our intra-domain task is triggering mainly neurocognitive operations that are devoted to the processing of the face structure.

In relation to the N170 elicited by occupations, the results allow us to establish a parallelism with the neural left-sided activity generated in the same latencies by common words (Rossion et al., 2003). Nevertheless, in the comparison of sources between N170 for faces and occupations, we have noted an enhanced right lingual activity for occupations vs. faces. In relation to the role that the lingual cortex might play in word processing, bilateral activity has been described in this posterior occipital region by Hagoort et al. (1999) using PET during silent reading of words in comparison with viewing a fixation cross. Moreover, Mechelli et al. (2000) used PET to assess the modulations of both fusiform and lingual gyri activations by word length and perceptive similarity to the background during reading. They found that both word length and visual contrast had a positive monotonic effect on activation in the bilateral fusiform region, however, the lingual gyrus activation increased specifically with increasing word length, but decreased with increasing contrast. These previous findings suggest that the comparatively larger lingual activity found here around 170 ms in the cross-domain task might reflect some low-level operation in verbal processing that occurs prior to word recognition. Speculatively, this might indicate that N170 for words might have a different functional role than the N170 elicited by faces.

### Right-Sided Occipital P200 and Temporal (Extensive Fusiform) N250 Were Only Elicited by Faces

An interesting finding of the present experiment is the exclusive elicitation of the P200 and N250 responses in our intra-domain task. The less studied P200 wave (Halit et al., 2000; Milivojevic et al., 2003; Boutsen et al., 2006; Schendan and Kutas, 2007; Lucas et al., 2011; Kaufmann and Schweinberger, 2012) seems to deal with the use of configural information to recognize individual faces since this positive peak, that appears in latencies some later than N170, is modulated, for example, by thatcherization and feature displacement in upright faces (Halit et al., 2000; Milivojevic et al., 2003; Boutsen et al., 2006; Lucas et al., 2011). In the present study, the sources for this early response were found in very posterior brain regions and a certain effect of lateralization was observed, which supports its role in both an early stage of visual perception and non-verbal (face) processing (Gainotti and Marra, 2011). As pointed out in the “Introduction” section, N250 (Begleiter et al., 1995; Schweinberger et al., 1995) is thought to reflect the access to or the transient activation of stored face representations. This right occipitotemporal response is characterized by more negative amplitudes for repeated (mainly familiar) than for non-repeated faces around 230–300 ms. Modulations of this component are found even with the presentation of different images of the same person, suggesting that it is related to the activation of relatively abstract representations concerning face structure, which are invariant over transformations of low-level visual cues (Schweinberger et al., 2002; Itier and Taylor, 2004; Boehm and Sommer, 2005). Schweinberger et al. (2007) observed that the reconstructed sources of the M250r, the neuromagnetic equivalent of N250r, were situated in the right fusiform region. The extensive and almost exclusive involvement of the right fusiform gyrus (encompassing the FFA and more anterior fusiform regions) and, to a lesser extent, parahippocampal regions in the sources of N250 in the present study, argues that this potential is clearly face-sensitive and reflects an ulterior stage in face processing to that represented by the face-sensitive N170. Accordingly, Grill-Spector et al. (2004) showed with fMRI that FFA activation was correlated on a trial-by-trial basis with both detecting the presence of faces and successful identification of specific famous faces, implicating this region in the extraction of information about face identity. All this points out that the relatively early responses P200 and N250, when elicited in identity-recognition experiments, may also constitute relevant neurophysiological markers for intra-domain (face) processing in contrast with face-related verbal information, insofar as both stimulus-associated knowledge and task demands can be strictly controlled.

### Reconstructed Neural Sources Reveal a Right Occipitotemporal Neural Origin for N400 for Faces and a More Left-Sided Temporal Origin for N400 for Occupations

A good deal of evidence in favor of differentiated neural systems for access to and retrieval from LTM of face structure and face-related verbal information in the present study is provided by source modeling of the elicited mismatch ERP effects. First, we found that reconstructed neural sources of the N400 for faces in the intra-domain task differed from those of the N400 for occupations primed by faces in the cross-domain task. In the case of faces, source modeling suggested a large contribution to this scalp ERP of the right inferior (predominantly) middle and superior occipital gyri, encompassing the OFA and also, to a great extent, the FFA. According to Pitcher et al. (2011), the OFA represents a part of the face-processing network engaged in componential face processing, which is prior to subsequent processing of increasingly visual complexity in higher face-selective cortical regions. Gilaie-Dotan et al. (2010), by using TMS, constated that both the OFA and the lateral occipital cortex were not affected by identity repetition of famous faces, thus suggesting that these regions may be involved in the processing of more generic facial features. Liu et al. (2010) used fMRI to measure the magnitude of response in the OFA, the FFA and the fSTS (a face-selective region in the Superior Temporal Sulcus) to stimuli that, first, either contained real face parts or did not, and second, either had veridical face configurations or did not. They reported that the OFA and the fSTS were only sensitive to the presence of real face parts, not to the correct configuration of those parts, whereas the FFA was sensitive to both face parts and face configuration. Furthermore, only in the FFA was the response to configuration and part information correlated across voxels, suggesting that the FFA contains a unified representation that includes both kinds of information. Accordingly, FFA has been found to respond to faces *per se* and not to lower level stimulus features usually present in faces (see Kanwisher and Yovel, 2006, for a review). These roles assigned to OFA and even to FFA in face processing and their observed involvement in the N400-like effect of our intra-domain task, suggest that, in spite of its relative long latency, our scalp response is essentially dealing with face processing. Furthermore, this also supports the notion that N400-like responses might reflect, according to task demands and stimulus properties, top-down perceptual mechanisms concerning visual recognition.

On the other hand, the reconstructed neural sources of the N400 for occupations in the cross-domain task closely resembled those reported in previous research regarding the N400 elicited by the processing of common words (see for example, Olichney et al., 2000; Halgren et al., 2002; Friederici et al., 2003; Bölte et al., 2010). Namely, in our cross-domain task mismatching occupations preceded by faces activated mostly left fusiform and temporal inferior regions (in addition to bilateral occipital inferior regions). The predominant left-sided activity found here, as well as the large coincidence of sources with the linguistic N400, shows that the processing of face-related verbal information might share, to a notable extent, the neural basis involved in the access and retrieval of general semantic memory and language. In addition, direct statistical comparison of intra-domain and cross-domain N400s in our study confirmed that both brain responses are differently lateralized, as well as that the former is likely dealing with perceptual processes concerning face structure, whereas the latter, with an enhanced anterior activity, is probably engaged in language-related ones.

It is important to note that all those laterality effects of possible neural generators of ERPs found in our study, denoting predominant right-sided sources in our intra-domain task and left-sided sources in the case of the cross-domain task, are in line with those findings described in the neuropsychological literature in relation with the type of identity-recognition disorders and the damaged hemisphere (see Gainotti, 2007; Gainotti and Marra, 2011, for reviews). Thus, when the lesion is right-sided a loss of both familiarity feelings and person-specific information retrieval from face stimuli are more commonly found whereas when the left hemisphere is affected, it exists a prevalent impairment in finding names or verbal information associated to known individuals. This could also explain why the predominantly right-sided P200 and N250 were exclusively elicited in our intra-domain task.

Summarizing, this ERP study has revealed that the access to and retrieval of face structural information can be differentiated, in terms of high-temporal resolution electrophysiological responses and their putative neural sources, from the processing of verbal information closely related to the faces and triggered by them. As expected, N400-like responses were elicited in both intra-domain and cross-domain tasks by incorrect eyes-eyebrows and occupations, respectively. Interestingly, source reconstruction indicated, in the case of the intra-domain N400, that its neural generators might be situated in right posterior brain cortices encompassing mainly OFA and, in some extent, FFA, thus reflecting perceptual operations triggered by physical incongruities. In turn, the N400 for occupations could be generated by more anterior left-sided fusiform and temporal inferior activity, paralleling both the activity described previously as accounting for classic verbal N400 and those neural sources proposed as being associated to person specific verbal information. Additionally, the earlier N170s generated in both cases were of similar amplitude but seemed to have different neural support, thus suggesting distinct functional roles in both cases. Whereas N170 for faces was principally associated to right fusiform and occipital activity, also involving face-related FFA and OFA areas, the N170 for occupations was predominantly associated to bilateral very posterior activity that denoted basic perceptual processes. Furthermore, the right-sided perceptual P200 and the face-related N250 components were elicited exclusively in the intra-domain task, with possible neural sources in OFA and extensive fusiform region, respectively. Thus, these two responses might constitute specific electrophysiological markers of face processing in different stages of the face-related brain network. All these results support the existence of differentiated neural subsystems for face structural and face-related verbal information processing, which can be activated according to the stimulus-associated knowledge and specific task demands.

### Methodological Remarks

From a methodological perspective, our study shows the pertinence of the application of massive univariate repeated measures ANOVAs to verify multiple experimental effects in ERPs as well as conjunction analyses of group data in source reconstruction research. These statistical tools and the application of Bayesian Model Averaging approach for source modeling, have allowed us to delineate the neural dynamic underlying face processing in contrast to the processing of verbal information associated with recently known faces. The decision to implement conjunction analyses in source modeling in this study was driven by the present limitations in group analysis of IS maps, which contains serious statistical complications. First, IS maps are a mixture of exactly zero values and coefficients from which a parametric model does not exist. Second, there are large variations in the scale of IS maps across participants. Consequently, there is no established method for second level (group) analysis of IS maps of reconstructed neural sources. Additionally, the estimation of a single IS map is a time-consuming computational task hence the use of permutations is not feasible. In the present study, to cope with these limitations, the conjunction analysis first extracted those voxels with higher contribution to the individual IS map and then estimated the conjunction of participants containing the same active voxel across the group (Friston et al., 1999).

In any event, despite of the potentially interesting results derived from the present study, we are cautious in terms of the degree of precision in the localization of neural sources that can be achieved from scalp-recorded data. Thus, new sources of evidence are necessary to cope with the actual limitations in neural source reconstruction in EEG and ERP studies. In this line, a relevant contribution to characterize the brain dynamics of both face and face-related verbal processing might be provided by “effective connectivity” analyses using the high temporal resolution of EEG and ERP data (Friston et al., 2003; David et al., 2006). This would help to delineate not only those specific nodes of activation but also their causal relationships in the face network of the brain.

On the other hand, in future experiments would be appropriate the use of photos of real faces as stimuli, since natural faces have relevant texture information which is lacking in line drawings of faces. Additionally, the use of occupations as primes and faces as targets in the cross-domain task would facilitate the comparison of physically identical target stimuli.

## Author Contributions

EIO has carried out the acquisition of data. EIO and JI have designed the study, analyzed the ERP data, interpreted the results and drafted the manuscript. AL-C has designed and carried out the statistical analysis of ERP and source reconstruction data as well as drafted the corresponding method section. MAB has contributed with relevant ideas to the interpretation of the results and has revised critically the manuscript. All authors agree to be accountable for the content of the work.

## Conflict of Interest Statement

The authors declare that the research was conducted in the absence of any commercial or financial relationships that could be construed as a potential conflict of interest.
